# (μ-4-Bromo-3,5-dimethyl­pyrazolato-κ^2^ *N* ^1^:*N* ^2^)-μ-chlorido-bis­[bis(4-bromo-3,5-dimethyl­pyrazole-κ*N* ^2^)chloridocopper(II)] acetonitrile monosolvate

**DOI:** 10.1107/S160053681201402X

**Published:** 2012-04-13

**Authors:** Wei Wei, Yanhui Xu

**Affiliations:** a Department of Chemistry, Capital Normal University, Beijing 100048, People’s Republic of China; b Department of Medical Imaging, Bethune Medical Non-Commissioned Officer’s, College, Shijiazhuang, Hebei 050081, People’s Republic of China

## Abstract

In the title dinuclear complex, [Cu_2_(C_5_H_6_BrN_2_)Cl_3_(C_5_H_7_BrN_2_)_4_]·CH_3_CN, both Cu^II^ ions are in slightly distorted square-pyramidal coordination geometries. The basal planes are defined by three N atoms from three 4-bromo-3,5-dimethyl­pyrazolate ligands, one of which is bridging, and one Cl ligand. A bridging Cl ligand forms the apical site for both Cu^II^ ions. In the crystal, N—H⋯Cl hydrogen bonds connect complex mol­ecules into chains along [100]. Intra­molecular N—H⋯Cl hydrogen bonds are also observed.

## Related literature

For related structures, see: Mezei & Raptis (2004 ▶).
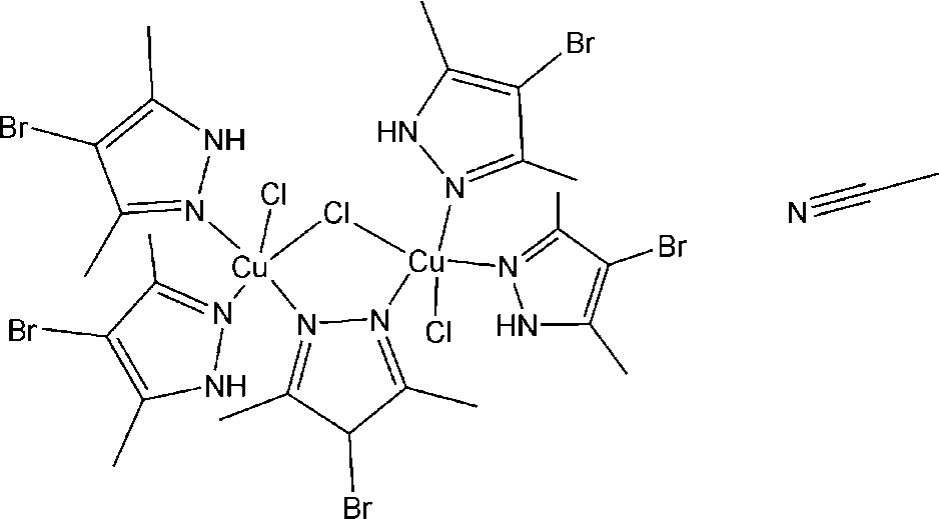


## Experimental

### 

#### Crystal data

[Cu_2_(C_5_H_6_BrN_2_)Cl_3_(C_5_H_7_BrN_2_)_4_]·CH_4_N
*M* *_r_* = 1148.66
Monoclinic, 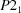

*a* = 9.282 (2) Å
*b* = 15.849 (4) Å
*c* = 14.711 (4) Å
β = 108.048 (4)°
*V* = 2057.5 (9) Å^3^
*Z* = 2
Mo *K*α radiation
μ = 6.12 mm^−1^
*T* = 93 K
0.33 × 0.30 × 0.27 mm

#### Data collection

Bruker SMART CCD diffractometer
Absorption correction: multi-scan (*SADABS*; Bruker, 2000 ▶) *T*_min_ = 0.235, *T*_max_ = 0.292
13972 measured reflections
6853 independent reflections
6098 reflections with *I* > 2σ(*I*)
*R*_int_ = 0.027

#### Refinement

*R*[*F* ^2^ > 2σ(*F* ^2^)] = 0.024
*wR*(*F* ^2^) = 0.036
*S* = 0.88
6853 reflections
444 parameters
1 restraint
H-atom parameters constrained
Δρ_max_ = 0.48 e Å^−3^
Δρ_min_ = −0.39 e Å^−3^
Absolute structure: Flack (1983 ▶), 3105 Friedel pairs
Flack parameter: 0.004 (5)

### 

Data collection: *SMART* (Bruker, 2000 ▶); cell refinement: *SAINT* (Bruker, 2000 ▶); data reduction: *SAINT*; program(s) used to solve structure: *SHELXTL* (Sheldrick, 2008 ▶); program(s) used to refine structure: *SHELXTL*; molecular graphics: *SHELXTL*; software used to prepare material for publication: *SHELXTL*.

## Supplementary Material

Crystal structure: contains datablock(s) I, global. DOI: 10.1107/S160053681201402X/lh5445sup1.cif

Structure factors: contains datablock(s) I. DOI: 10.1107/S160053681201402X/lh5445Isup2.hkl

Additional supplementary materials:  crystallographic information; 3D view; checkCIF report

## Figures and Tables

**Table 1:** Hydrogen-bond geometry (Å, °)

*D*—H⋯*A*	*D*—H	H⋯*A*	*D*⋯*A*	*D*—H⋯*A*
N4—H4*N*⋯Cl3^i^	0.88	2.36	3.194 (4)	160
N10—H10*N*⋯Cl2^ii^	0.88	2.33	3.144 (3)	155
N2—H2*N*⋯Cl3	0.88	2.54	3.400 (4)	165
N8—H8*N*⋯Cl2	0.88	2.34	3.212 (3)	170

Symmetry codes: (i) 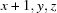; (ii) 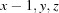.

**Fig. 1.**
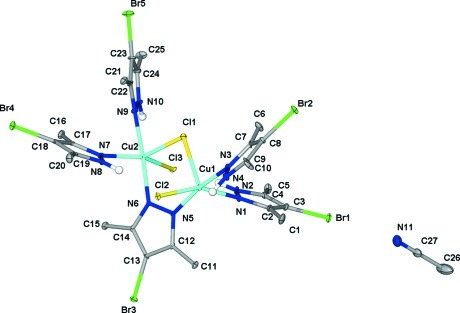
The molecular structure of the title compound with displacement ellipsoids drawn at the 30% probability level.

**Fig. 2.**
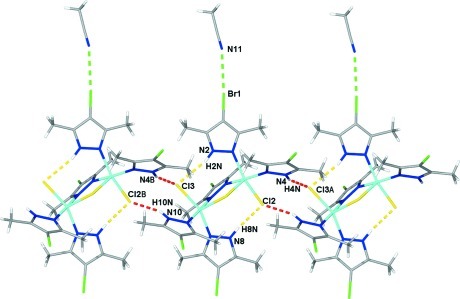
Part of the crystal structure showing a one-dimensional chain assembled by intermolecular N—H···Cl hydrogen bonds (dashed lines). The symmetry codes are: (A) 1+x,y, z; (B) x-1, y, z.

## supplementary crystallographic information

### Comment

The title binuclear complex is related to structures published by Mezei & Raptis (2004). The molecular structure of the title complex is shown in Fig. 1. Each Cu^II^ ion is five-coordinated, adopting a slight distorted square-pyramidal coordination geometry defined by three nitrogen atoms from three 4-bromo-3,5-dimethylpyrazolato (bdpz) ligands and two Cl ligands. The two Cu^II^ ions are linked *via* one *µ*_2_-Cl ligand and two nitrogen atoms from a *µ*_2_-bdpz ligand, giving a five-membered Cu_2_N_2_Cl ring. The bdpz ligands have two different coordination modes. One is monodentate and the other is bidentate. The bidentated bridge ligand is depronated so that both of the nitrogen atoms are able to coordinate to two Cu^II^ ions to form a binuclear structure with a Cu1···Cu2 distance of 3.6874 (10) Å. The monodentate bdpz ligands coordinate through one nitrogen atom each. The two Cu^II^ ions are charge balanced by the three Cl^-^ ligands and one deprotaonted bdpz ligand. In the crystal, symmetry related complex molecules are connected bdpz via N—H···Cl hydrogen bonds (Table 1) to form a 1-D supramolecular structure (Fig 2). In addition, intramolecular N—H···O hydrogen bonds are present and it is worthy of note that a short C—Br···N contact exists (Br···N11 = 3.089 (2) Å).

### Experimental

CuCl_2._2H_2_O (0.170 g, 1 mmol) and bdpz (0.131 g, 0.75 mmol) were dissolved in 15 ml acetonitrile and stirred for ten minutes at room temprature. Then the mixture were transfered to a Teflon container and heated at 363K for 48 h. Green block-shaped crystals suitable for X-ray diffraction analysis were obtained after filtration. Yield: about 18% (based on the bdpz).

### Refinement

Hydrogen atoms positions were calculated and refined in a riding-model approxination with *U*_iso_(H) = 1.2U_eq_(C,N).

### Crystal data

**Table:**

[Cu_2_(C_5_H_6_BrN_2_)Cl_3_(C_5_H_7_BrN_2_)_4_]·CH_4_N	*F*(000) = 1120
*M**_r_* = 1148.66	*D*_x_ = 1.854 Mg m^−^^3^
Monoclinic, *P*2_1_	Mo *K*α radiation, λ = 0.71073 Å
Hall symbol: P 2yb	Cell parameters from 1336 reflections
*a* = 9.282 (2) Å	θ = 2.6–27.5°
*b* = 15.849 (4) Å	µ = 6.12 mm^−^^1^
*c* = 14.711 (4) Å	*T* = 93 K
β = 108.048 (4)°	Block, green
*V* = 2057.5 (9) Å^3^	0.33 × 0.30 × 0.27 mm
*Z* = 2	

### Data collection

**Table:**

Bruker SMART CCD diffractometer	6853 independent reflections
Radiation source: fine-focus sealed tube	6098 reflections with *I* > 2σ(*I*)
Graphite monochromator	*R*_int_ = 0.027
φ and ω scans	θ_max_ = 25.0°, θ_min_ = 3.1°
Absorption correction: multi-scan (*SADABS*; Bruker, 2000)	*h* = −11→9
*T*_min_ = 0.235, *T*_max_ = 0.292	*k* = −14→18
13972 measured reflections	*l* = −17→17

### Refinement

**Table:**

Refinement on *F*^2^	Secondary atom site location: difference Fourier map
Least-squares matrix: full	Hydrogen site location: inferred from neighbouring sites
*R*[*F*^2^ > 2σ(*F*^2^)] = 0.024	H-atom parameters constrained
*wR*(*F*^2^) = 0.036	[1.00000 + 0.00000exp(0.00(sinθ/λ)^2^)]/ [σ^2^(*F*_o_^2^) + 0.0000 + 0.0000**P* + (0.0021*P*)^2^ + 0.0000sinθ/λ] where *P* = 0.00000*F*_o_^2^ + 1.00000*F*_c_^2^
*S* = 0.88	(Δ/σ)_max_ = 0.003
6853 reflections	Δρ_max_ = 0.48 e Å^−^^3^
444 parameters	Δρ_min_ = −0.39 e Å^−^^3^
1 restraint	Absolute structure: Flack (1983), 3105 Friedel pairs
Primary atom site location: structure-invariant direct methods	Flack parameter: 0.004 (5)

### Special details

**Table:**

Geometry. All e.s.d.'s (except the e.s.d. in the dihedral angle between two l.s. planes) are estimated using the full covariance matrix. The cell e.s.d.'s are taken into account individually in the estimation of e.s.d.'s in distances, angles and torsion angles; correlations between e.s.d.'s in cell parameters are only used when they are defined by crystal symmetry. An approximate (isotropic) treatment of cell e.s.d.'s is used for estimating e.s.d.'s involving l.s. planes.
Refinement. Refinement of *F*^2^ against ALL reflections. The weighted *R*-factor *wR* and goodness of fit *S* are based on *F*^2^, conventional *R*-factors *R* are based on *F*, with *F* set to zero for negative *F*^2^. The threshold expression of *F*^2^ > σ(*F*^2^) is used only for calculating *R*-factors(gt) *etc*. and is not relevant to the choice of reflections for refinement. *R*-factors based on *F*^2^ are statistically about twice as large as those based on *F*, and *R*- factors based on ALL data will be even larger.

### Fractional atomic coordinates and isotropic or equivalent isotropic displacement parameters (Å^2^)

**Table:**

	*x*	*y*	*z*	*U*_iso_*/*U*_eq_	
Cu1	0.68535 (6)	0.38846 (3)	0.63798 (4)	0.01178 (12)	
Cu2	0.44108 (6)	0.40441 (3)	0.79285 (4)	0.01135 (12)	
Br1	0.30510 (5)	0.30904 (3)	0.23256 (3)	0.02427 (12)	
Br2	0.83209 (5)	0.66236 (3)	0.40460 (3)	0.02460 (12)	
Br3	0.64363 (5)	0.05276 (3)	0.78378 (3)	0.02291 (12)	
Br4	0.79097 (6)	0.48030 (3)	1.19888 (4)	0.03071 (13)	
Br5	0.24518 (6)	0.74934 (3)	0.87086 (4)	0.03359 (14)	
Cl1	0.54114 (12)	0.49340 (6)	0.69126 (7)	0.0149 (2)	
Cl2	0.90648 (11)	0.37929 (6)	0.76878 (8)	0.0146 (3)	
Cl3	0.21862 (11)	0.35091 (6)	0.68680 (8)	0.0139 (2)	
N1	0.5288 (4)	0.3545 (2)	0.5111 (3)	0.0128 (8)	
N2	0.3830 (4)	0.3507 (2)	0.5099 (3)	0.0172 (9)	
H2N	0.3546	0.3574	0.5612	0.021*	
N3	0.7845 (4)	0.4692 (2)	0.5715 (2)	0.0152 (8)	
N4	0.9346 (4)	0.4677 (2)	0.5834 (2)	0.0160 (9)	
H4N	0.9976	0.4315	0.6207	0.019*	
N5	0.6118 (4)	0.29498 (19)	0.6978 (2)	0.0101 (8)	
N6	0.5436 (3)	0.30017 (19)	0.7671 (2)	0.0086 (8)	
N7	0.5978 (4)	0.41246 (19)	0.9240 (2)	0.0110 (8)	
N8	0.7457 (4)	0.41968 (19)	0.9283 (2)	0.0138 (8)	
H8N	0.7794	0.4109	0.8795	0.017*	
N9	0.3306 (4)	0.50622 (19)	0.8246 (3)	0.0138 (9)	
N10	0.1781 (4)	0.50677 (19)	0.8051 (2)	0.0147 (9)	
H10N	0.1202	0.4621	0.7864	0.018*	
C1	0.6637 (5)	0.3400 (3)	0.3906 (3)	0.0309 (13)	
H1A	0.7508	0.3265	0.4463	0.037*	
H1B	0.6786	0.3956	0.3657	0.037*	
H1C	0.6541	0.2973	0.3410	0.037*	
C2	0.5228 (5)	0.3410 (3)	0.4194 (3)	0.0181 (11)	
C3	0.3752 (5)	0.3290 (2)	0.3633 (3)	0.0179 (11)	
C4	0.2858 (5)	0.3356 (2)	0.4227 (3)	0.0158 (11)	
C5	0.1197 (5)	0.3286 (3)	0.4027 (3)	0.0281 (12)	
H5A	0.0881	0.2701	0.3862	0.034*	
H5B	0.0682	0.3656	0.3491	0.034*	
H5C	0.0929	0.3454	0.4595	0.034*	
C6	0.5694 (5)	0.5604 (3)	0.4809 (3)	0.0288 (12)	
H6A	0.5436	0.5819	0.5364	0.035*	
H6B	0.5043	0.5121	0.4536	0.035*	
H6C	0.5538	0.6050	0.4326	0.035*	
C7	0.7300 (5)	0.5335 (3)	0.5110 (3)	0.0149 (10)	
C8	0.8482 (5)	0.5690 (3)	0.4860 (3)	0.0150 (10)	
C9	0.9774 (5)	0.5268 (2)	0.5328 (3)	0.0164 (11)	
C10	1.1380 (5)	0.5415 (3)	0.5360 (3)	0.0278 (13)	
H10A	1.1534	0.5196	0.4774	0.033*	
H10B	1.2062	0.5123	0.5915	0.033*	
H10C	1.1597	0.6021	0.5413	0.033*	
C11	0.7424 (5)	0.1886 (2)	0.6275 (3)	0.0168 (11)	
H11A	0.8306	0.2255	0.6370	0.020*	
H11B	0.6769	0.1930	0.5611	0.020*	
H11C	0.7764	0.1301	0.6413	0.020*	
C12	0.6563 (5)	0.2149 (2)	0.6930 (3)	0.0127 (10)	
C13	0.6099 (5)	0.1685 (2)	0.7602 (3)	0.0123 (10)	
C14	0.5387 (5)	0.2238 (2)	0.8043 (3)	0.0117 (10)	
C15	0.4627 (5)	0.2064 (3)	0.8781 (3)	0.0219 (11)	
H15A	0.4191	0.2587	0.8935	0.026*	
H15B	0.5370	0.1841	0.9359	0.026*	
H15C	0.3819	0.1648	0.8532	0.026*	
C16	0.4480 (5)	0.4266 (3)	1.0340 (3)	0.0247 (12)	
H16A	0.3745	0.3923	0.9861	0.030*	
H16B	0.4076	0.4837	1.0343	0.030*	
H16C	0.4666	0.4010	1.0973	0.030*	
C17	0.5926 (5)	0.4307 (2)	1.0104 (3)	0.0135 (10)	
C18	0.7366 (5)	0.4500 (2)	1.0693 (3)	0.0161 (11)	
C19	0.8344 (5)	0.4419 (2)	1.0159 (3)	0.0158 (11)	
C20	1.0009 (5)	0.4534 (3)	1.0394 (3)	0.0265 (12)	
H20A	1.0260	0.5133	1.0515	0.032*	
H20B	1.0336	0.4341	0.9857	0.032*	
H20C	1.0528	0.4205	1.0966	0.032*	
C21	0.5369 (5)	0.6136 (3)	0.8794 (3)	0.0230 (12)	
H21A	0.5912	0.5838	0.8416	0.028*	
H21B	0.5848	0.6014	0.9474	0.028*	
H21C	0.5404	0.6745	0.8685	0.028*	
C22	0.3763 (5)	0.5851 (2)	0.8501 (3)	0.0146 (11)	
C23	0.2503 (5)	0.6333 (2)	0.8453 (3)	0.0178 (11)	
C24	0.1258 (5)	0.5825 (3)	0.8175 (3)	0.0211 (11)	
C25	−0.0401 (5)	0.6002 (3)	0.7997 (3)	0.0304 (13)	
H25A	−0.0908	0.5486	0.8103	0.036*	
H25B	−0.0856	0.6192	0.7335	0.036*	
H25C	−0.0518	0.6443	0.8435	0.036*	
N11	0.1755 (5)	0.2600 (3)	0.0195 (3)	0.0448 (12)	
C26	0.0325 (7)	0.1850 (3)	−0.1368 (5)	0.085 (2)	
H26A	0.0798	0.1300	−0.1390	0.102*	
H26B	0.0386	0.2196	−0.1907	0.102*	
H26C	−0.0740	0.1768	−0.1409	0.102*	
C27	0.1126 (6)	0.2277 (3)	−0.0463 (4)	0.0346 (14)	

### Atomic displacement parameters (Å^2^)

**Table:**

	*U*^11^	*U*^22^	*U*^33^	*U*^12^	*U*^13^	*U*^23^
Cu1	0.0109 (3)	0.0159 (3)	0.0089 (3)	−0.0013 (2)	0.0036 (2)	0.0016 (2)
Cu2	0.0110 (3)	0.0130 (3)	0.0103 (3)	0.0000 (2)	0.0038 (2)	−0.0026 (2)
Br1	0.0301 (3)	0.0309 (3)	0.0095 (3)	−0.0036 (2)	0.0028 (2)	−0.0027 (2)
Br2	0.0291 (3)	0.0217 (3)	0.0235 (3)	0.0028 (2)	0.0088 (2)	0.0140 (2)
Br3	0.0333 (3)	0.0139 (2)	0.0234 (3)	0.0052 (2)	0.0115 (3)	0.0051 (2)
Br4	0.0456 (4)	0.0299 (3)	0.0108 (3)	0.0007 (3)	0.0002 (3)	−0.0047 (2)
Br5	0.0331 (3)	0.0134 (3)	0.0488 (4)	0.0019 (2)	0.0047 (3)	−0.0100 (3)
Cl1	0.0175 (6)	0.0137 (6)	0.0159 (6)	0.0007 (5)	0.0087 (5)	0.0013 (5)
Cl2	0.0103 (6)	0.0209 (6)	0.0116 (6)	−0.0028 (5)	0.0020 (5)	0.0036 (5)
Cl3	0.0080 (6)	0.0159 (6)	0.0154 (6)	0.0014 (5)	0.0002 (5)	−0.0077 (5)
N1	0.007 (2)	0.022 (2)	0.010 (2)	−0.0004 (16)	0.0036 (18)	0.0020 (16)
N2	0.020 (2)	0.025 (2)	0.009 (2)	−0.0033 (18)	0.0074 (19)	−0.0008 (17)
N3	0.013 (2)	0.023 (2)	0.011 (2)	−0.0022 (17)	0.0058 (18)	0.0026 (17)
N4	0.009 (2)	0.022 (2)	0.016 (2)	0.0066 (17)	0.0026 (18)	0.0076 (17)
N5	0.0070 (19)	0.012 (2)	0.010 (2)	0.0009 (15)	0.0021 (17)	−0.0060 (16)
N6	0.0056 (19)	0.0113 (19)	0.008 (2)	−0.0024 (16)	0.0005 (16)	−0.0008 (16)
N7	0.0064 (19)	0.013 (2)	0.014 (2)	−0.0004 (15)	0.0039 (17)	−0.0032 (16)
N8	0.017 (2)	0.015 (2)	0.010 (2)	−0.0005 (16)	0.0048 (18)	−0.0008 (16)
N9	0.013 (2)	0.012 (2)	0.018 (2)	−0.0016 (16)	0.0078 (19)	−0.0027 (16)
N10	0.008 (2)	0.014 (2)	0.020 (2)	−0.0045 (16)	0.0023 (18)	−0.0077 (16)
C1	0.033 (3)	0.047 (3)	0.017 (3)	0.002 (3)	0.015 (3)	−0.005 (3)
C2	0.024 (3)	0.020 (3)	0.011 (3)	0.003 (2)	0.006 (2)	−0.002 (2)
C3	0.025 (3)	0.017 (3)	0.010 (3)	−0.008 (2)	0.003 (2)	−0.005 (2)
C4	0.020 (3)	0.016 (3)	0.010 (3)	0.000 (2)	0.003 (2)	0.0018 (19)
C5	0.021 (3)	0.042 (3)	0.021 (3)	0.000 (2)	0.006 (3)	0.002 (2)
C6	0.021 (3)	0.037 (3)	0.029 (3)	0.012 (2)	0.009 (2)	0.018 (3)
C7	0.015 (3)	0.022 (3)	0.004 (2)	0.005 (2)	−0.001 (2)	0.005 (2)
C8	0.016 (3)	0.015 (2)	0.016 (3)	0.003 (2)	0.007 (2)	0.005 (2)
C9	0.018 (3)	0.011 (2)	0.021 (3)	0.0001 (19)	0.007 (2)	0.0111 (19)
C10	0.022 (3)	0.029 (3)	0.041 (3)	0.010 (2)	0.022 (3)	0.024 (3)
C11	0.017 (3)	0.021 (3)	0.014 (3)	−0.005 (2)	0.008 (2)	−0.005 (2)
C12	0.011 (2)	0.018 (3)	0.008 (3)	−0.0029 (19)	0.001 (2)	−0.007 (2)
C13	0.014 (2)	0.007 (2)	0.012 (2)	−0.002 (2)	−0.001 (2)	0.004 (2)
C14	0.009 (2)	0.018 (3)	0.007 (2)	−0.0013 (19)	0.001 (2)	−0.0011 (19)
C15	0.029 (3)	0.024 (2)	0.021 (3)	0.001 (2)	0.021 (2)	0.001 (2)
C16	0.033 (3)	0.029 (3)	0.020 (3)	−0.002 (2)	0.018 (3)	−0.002 (2)
C17	0.016 (3)	0.009 (2)	0.016 (3)	0.0039 (19)	0.007 (2)	0.000 (2)
C18	0.031 (3)	0.007 (2)	0.009 (3)	0.001 (2)	0.004 (2)	−0.0002 (19)
C19	0.017 (3)	0.006 (2)	0.018 (3)	0.0012 (19)	−0.004 (2)	0.001 (2)
C20	0.023 (3)	0.035 (3)	0.018 (3)	−0.002 (2)	0.002 (2)	0.001 (2)
C21	0.026 (3)	0.024 (3)	0.020 (3)	−0.004 (2)	0.008 (3)	−0.006 (2)
C22	0.021 (3)	0.012 (2)	0.013 (3)	−0.005 (2)	0.009 (2)	−0.0044 (19)
C23	0.019 (3)	0.007 (2)	0.024 (3)	0.002 (2)	0.003 (2)	−0.003 (2)
C24	0.023 (3)	0.017 (3)	0.025 (3)	0.006 (2)	0.010 (2)	0.000 (2)
C25	0.011 (3)	0.029 (3)	0.045 (4)	0.005 (2)	0.000 (3)	−0.009 (3)
N11	0.046 (3)	0.052 (3)	0.030 (3)	0.008 (3)	0.002 (3)	0.003 (3)
C26	0.080 (6)	0.042 (4)	0.095 (6)	0.006 (3)	−0.028 (5)	−0.011 (4)
C27	0.035 (4)	0.029 (3)	0.027 (4)	0.004 (2)	−0.009 (3)	−0.001 (3)

### Geometric parameters (Å, º)

**Table:**

Cu1—N5	1.951 (3)	C6—H6A	0.9800
Cu1—N3	1.999 (3)	C6—H6B	0.9800
Cu1—N1	2.051 (4)	C6—H6C	0.9800
Cu1—Cl2	2.3429 (13)	C7—C8	1.381 (5)
Cu1—Cl1	2.4128 (11)	C8—C9	1.360 (5)
Cu2—N6	2.000 (3)	C9—C10	1.495 (5)
Cu2—N7	2.028 (3)	C10—H10A	0.9800
Cu2—N9	2.042 (3)	C10—H10B	0.9800
Cu2—Cl3	2.3281 (12)	C10—H10C	0.9800
Cu2—Cl1	2.4382 (11)	C11—C12	1.490 (5)
Br1—C3	1.857 (4)	C11—H11A	0.9800
Br2—C8	1.880 (4)	C11—H11B	0.9800
Br3—C13	1.875 (4)	C11—H11C	0.9800
Br4—C18	1.876 (4)	C12—C13	1.403 (5)
Br5—C23	1.881 (4)	C13—C14	1.375 (5)
N1—C2	1.349 (5)	C14—C15	1.492 (5)
N1—N2	1.349 (4)	C15—H15A	0.9800
N2—C4	1.342 (5)	C15—H15B	0.9800
N2—H2N	0.8800	C15—H15C	0.9800
N3—C7	1.345 (5)	C16—C17	1.489 (5)
N3—N4	1.349 (4)	C16—H16A	0.9800
N4—C9	1.331 (4)	C16—H16B	0.9800
N4—H4N	0.8800	C16—H16C	0.9800
N5—C12	1.343 (5)	C17—C18	1.384 (6)
N5—N6	1.359 (4)	C18—C19	1.379 (6)
N6—C14	1.334 (5)	C19—C20	1.486 (6)
N7—C17	1.319 (5)	C20—H20A	0.9800
N7—N8	1.359 (4)	C20—H20B	0.9800
N8—C19	1.343 (5)	C20—H20C	0.9800
N8—H8N	0.8800	C21—C22	1.488 (5)
N9—C22	1.335 (5)	C21—H21A	0.9800
N9—N10	1.355 (4)	C21—H21B	0.9800
N10—C24	1.329 (5)	C21—H21C	0.9800
N10—H10N	0.8800	C22—C23	1.381 (5)
C1—C2	1.494 (5)	C23—C24	1.362 (6)
C1—H1A	0.9800	C24—C25	1.506 (5)
C1—H1B	0.9800	C25—H25A	0.9800
C1—H1C	0.9800	C25—H25B	0.9800
C2—C3	1.376 (6)	C25—H25C	0.9800
C3—C4	1.383 (5)	N11—C27	1.090 (6)
C4—C5	1.481 (5)	C26—C27	1.473 (7)
C5—H5A	0.9800	C26—H26A	0.9800
C5—H5B	0.9800	C26—H26B	0.9800
C5—H5C	0.9800	C26—H26C	0.9800
C6—C7	1.480 (5)		
			
N5—Cu1—N3	169.97 (13)	C9—C8—Br2	126.4 (3)
N5—Cu1—N1	87.87 (13)	C7—C8—Br2	125.3 (3)
N3—Cu1—N1	91.52 (13)	N4—C9—C8	105.1 (4)
N5—Cu1—Cl2	85.56 (10)	N4—C9—C10	124.0 (4)
N3—Cu1—Cl2	91.28 (11)	C8—C9—C10	130.8 (4)
N1—Cu1—Cl2	157.62 (9)	C9—C10—H10A	109.5
N5—Cu1—Cl1	94.01 (9)	C9—C10—H10B	109.5
N3—Cu1—Cl1	95.96 (10)	H10A—C10—H10B	109.5
N1—Cu1—Cl1	99.72 (9)	C9—C10—H10C	109.5
Cl2—Cu1—Cl1	102.06 (4)	H10A—C10—H10C	109.5
N6—Cu2—N7	89.37 (13)	H10B—C10—H10C	109.5
N6—Cu2—N9	176.29 (13)	C12—C11—H11A	109.5
N7—Cu2—N9	89.75 (13)	C12—C11—H11B	109.5
N6—Cu2—Cl3	86.99 (10)	H11A—C11—H11B	109.5
N7—Cu2—Cl3	152.38 (10)	C12—C11—H11C	109.5
N9—Cu2—Cl3	92.13 (11)	H11A—C11—H11C	109.5
N6—Cu2—Cl1	93.89 (9)	H11B—C11—H11C	109.5
N7—Cu2—Cl1	104.44 (10)	N5—C12—C13	106.9 (3)
N9—Cu2—Cl1	89.81 (9)	N5—C12—C11	122.3 (4)
Cl3—Cu2—Cl1	103.13 (4)	C13—C12—C11	130.8 (4)
Cu1—Cl1—Cu2	98.94 (4)	C14—C13—C12	107.2 (4)
C2—N1—N2	104.5 (4)	C14—C13—Br3	127.9 (3)
C2—N1—Cu1	138.6 (3)	C12—C13—Br3	125.0 (3)
N2—N1—Cu1	116.6 (3)	N6—C14—C13	107.6 (3)
C4—N2—N1	113.2 (3)	N6—C14—C15	123.4 (4)
C4—N2—H2N	123.4	C13—C14—C15	129.0 (4)
N1—N2—H2N	123.4	C14—C15—H15A	109.5
C7—N3—N4	105.1 (3)	C14—C15—H15B	109.5
C7—N3—Cu1	132.3 (3)	H15A—C15—H15B	109.5
N4—N3—Cu1	122.6 (3)	C14—C15—H15C	109.5
C9—N4—N3	113.0 (3)	H15A—C15—H15C	109.5
C9—N4—H4N	123.5	H15B—C15—H15C	109.5
N3—N4—H4N	123.5	C17—C16—H16A	109.5
C12—N5—N6	108.6 (3)	C17—C16—H16B	109.5
C12—N5—Cu1	122.5 (3)	H16A—C16—H16B	109.5
N6—N5—Cu1	127.0 (2)	C17—C16—H16C	109.5
C14—N6—N5	109.6 (3)	H16A—C16—H16C	109.5
C14—N6—Cu2	126.3 (3)	H16B—C16—H16C	109.5
N5—N6—Cu2	123.5 (2)	N7—C17—C18	109.6 (4)
C17—N7—N8	105.9 (3)	N7—C17—C16	121.3 (4)
C17—N7—Cu2	134.5 (3)	C18—C17—C16	129.0 (4)
N8—N7—Cu2	117.8 (2)	C19—C18—C17	107.5 (4)
C19—N8—N7	112.2 (3)	C19—C18—Br4	125.9 (4)
C19—N8—H8N	123.9	C17—C18—Br4	126.6 (4)
N7—N8—H8N	123.9	N8—C19—C18	104.8 (4)
C22—N9—N10	105.9 (3)	N8—C19—C20	122.7 (4)
C22—N9—Cu2	131.7 (3)	C18—C19—C20	132.5 (4)
N10—N9—Cu2	121.5 (2)	C19—C20—H20A	109.5
C24—N10—N9	112.3 (3)	C19—C20—H20B	109.5
C24—N10—H10N	123.9	H20A—C20—H20B	109.5
N9—N10—H10N	123.9	C19—C20—H20C	109.5
C2—C1—H1A	109.5	H20A—C20—H20C	109.5
C2—C1—H1B	109.5	H20B—C20—H20C	109.5
H1A—C1—H1B	109.5	C22—C21—H21A	109.5
C2—C1—H1C	109.5	C22—C21—H21B	109.5
H1A—C1—H1C	109.5	H21A—C21—H21B	109.5
H1B—C1—H1C	109.5	C22—C21—H21C	109.5
N1—C2—C3	110.3 (4)	H21A—C21—H21C	109.5
N1—C2—C1	121.1 (4)	H21B—C21—H21C	109.5
C3—C2—C1	128.7 (4)	N9—C22—C23	108.3 (4)
C2—C3—C4	107.0 (4)	N9—C22—C21	124.6 (4)
C2—C3—Br1	127.5 (3)	C23—C22—C21	127.1 (4)
C4—C3—Br1	125.5 (4)	C24—C23—C22	108.3 (4)
N2—C4—C3	105.1 (4)	C24—C23—Br5	124.5 (3)
N2—C4—C5	123.6 (4)	C22—C23—Br5	127.2 (3)
C3—C4—C5	131.3 (4)	N10—C24—C23	105.2 (4)
C4—C5—H5A	109.5	N10—C24—C25	122.7 (4)
C4—C5—H5B	109.5	C23—C24—C25	132.0 (4)
H5A—C5—H5B	109.5	C24—C25—H25A	109.5
C4—C5—H5C	109.5	C24—C25—H25B	109.5
H5A—C5—H5C	109.5	H25A—C25—H25B	109.5
H5B—C5—H5C	109.5	C24—C25—H25C	109.5
C7—C6—H6A	109.5	H25A—C25—H25C	109.5
C7—C6—H6B	109.5	H25B—C25—H25C	109.5
H6A—C6—H6B	109.5	C27—C26—H26A	109.5
C7—C6—H6C	109.5	C27—C26—H26B	109.5
H6A—C6—H6C	109.5	H26A—C26—H26B	109.5
H6B—C6—H6C	109.5	C27—C26—H26C	109.5
N3—C7—C8	108.6 (4)	H26A—C26—H26C	109.5
N3—C7—C6	123.7 (4)	H26B—C26—H26C	109.5
C8—C7—C6	127.8 (4)	N11—C27—C26	177.7 (7)
C9—C8—C7	108.2 (4)		
			
N5—Cu1—Cl1—Cu2	6.36 (10)	N1—C2—C3—C4	0.2 (5)
N3—Cu1—Cl1—Cu2	−172.55 (10)	C1—C2—C3—C4	179.8 (4)
N1—Cu1—Cl1—Cu2	94.88 (10)	N1—C2—C3—Br1	179.5 (3)
Cl2—Cu1—Cl1—Cu2	−79.95 (5)	C1—C2—C3—Br1	−0.9 (7)
N6—Cu2—Cl1—Cu1	0.07 (10)	N1—N2—C4—C3	0.1 (5)
N7—Cu2—Cl1—Cu1	90.42 (10)	N1—N2—C4—C5	−179.9 (3)
N9—Cu2—Cl1—Cu1	−179.89 (11)	C2—C3—C4—N2	−0.2 (5)
Cl3—Cu2—Cl1—Cu1	−87.74 (5)	Br1—C3—C4—N2	−179.5 (3)
N5—Cu1—N1—C2	−132.4 (4)	C2—C3—C4—C5	179.9 (4)
N3—Cu1—N1—C2	37.5 (4)	Br1—C3—C4—C5	0.6 (7)
Cl2—Cu1—N1—C2	−59.5 (5)	N4—N3—C7—C8	1.0 (5)
Cl1—Cu1—N1—C2	133.9 (4)	Cu1—N3—C7—C8	180.0 (3)
N5—Cu1—N1—N2	55.9 (3)	N4—N3—C7—C6	−177.5 (4)
N3—Cu1—N1—N2	−134.2 (3)	Cu1—N3—C7—C6	1.5 (7)
Cl2—Cu1—N1—N2	128.8 (2)	N3—C7—C8—C9	−0.9 (5)
Cl1—Cu1—N1—N2	−37.9 (3)	C6—C7—C8—C9	177.5 (4)
C2—N1—N2—C4	0.0 (4)	N3—C7—C8—Br2	−178.8 (3)
Cu1—N1—N2—C4	174.3 (3)	C6—C7—C8—Br2	−0.5 (7)
N5—Cu1—N3—C7	140.2 (7)	N3—N4—C9—C8	0.3 (5)
N1—Cu1—N3—C7	53.8 (4)	N3—N4—C9—C10	177.3 (4)
Cl2—Cu1—N3—C7	−148.4 (4)	C7—C8—C9—N4	0.4 (5)
Cl1—Cu1—N3—C7	−46.1 (4)	Br2—C8—C9—N4	178.3 (3)
N5—Cu1—N3—N4	−41.0 (10)	C7—C8—C9—C10	−176.4 (4)
N1—Cu1—N3—N4	−127.4 (3)	Br2—C8—C9—C10	1.6 (8)
Cl2—Cu1—N3—N4	30.4 (3)	N6—N5—C12—C13	1.7 (4)
Cl1—Cu1—N3—N4	132.7 (3)	Cu1—N5—C12—C13	167.0 (3)
C7—N3—N4—C9	−0.8 (4)	N6—N5—C12—C11	−176.7 (4)
Cu1—N3—N4—C9	−179.9 (3)	Cu1—N5—C12—C11	−11.4 (6)
N3—Cu1—N5—C12	−5.8 (10)	N5—C12—C13—C14	−0.5 (5)
N1—Cu1—N5—C12	80.9 (3)	C11—C12—C13—C14	177.8 (4)
Cl2—Cu1—N5—C12	−77.7 (3)	N5—C12—C13—Br3	−179.8 (3)
Cl1—Cu1—N5—C12	−179.5 (3)	C11—C12—C13—Br3	−1.6 (7)
N3—Cu1—N5—N6	156.7 (7)	N5—N6—C14—C13	2.0 (5)
N1—Cu1—N5—N6	−116.6 (3)	Cu2—N6—C14—C13	173.5 (3)
Cl2—Cu1—N5—N6	84.8 (3)	N5—N6—C14—C15	−176.2 (4)
Cl1—Cu1—N5—N6	−17.0 (3)	Cu2—N6—C14—C15	−4.7 (6)
C12—N5—N6—C14	−2.4 (4)	C12—C13—C14—N6	−1.0 (5)
Cu1—N5—N6—C14	−166.8 (3)	Br3—C13—C14—N6	178.4 (3)
C12—N5—N6—Cu2	−174.1 (3)	C12—C13—C14—C15	177.1 (4)
Cu1—N5—N6—Cu2	21.5 (4)	Br3—C13—C14—C15	−3.6 (7)
N7—Cu2—N6—C14	74.1 (3)	N8—N7—C17—C18	0.4 (4)
Cl3—Cu2—N6—C14	−78.5 (3)	Cu2—N7—C17—C18	−163.3 (3)
Cl1—Cu2—N6—C14	178.5 (3)	N8—N7—C17—C16	−177.9 (3)
N7—Cu2—N6—N5	−115.6 (3)	Cu2—N7—C17—C16	18.4 (6)
Cl3—Cu2—N6—N5	91.8 (3)	N7—C17—C18—C19	−0.8 (5)
Cl1—Cu2—N6—N5	−11.1 (3)	C16—C17—C18—C19	177.3 (4)
N6—Cu2—N7—C17	−138.3 (4)	N7—C17—C18—Br4	179.9 (3)
N9—Cu2—N7—C17	38.1 (4)	C16—C17—C18—Br4	−2.0 (6)
Cl3—Cu2—N7—C17	−56.0 (5)	N7—N8—C19—C18	−0.7 (4)
Cl1—Cu2—N7—C17	127.8 (4)	N7—N8—C19—C20	179.7 (3)
N6—Cu2—N7—N8	59.5 (3)	C17—C18—C19—N8	0.9 (4)
N9—Cu2—N7—N8	−124.1 (3)	Br4—C18—C19—N8	−179.8 (3)
Cl3—Cu2—N7—N8	141.8 (2)	C17—C18—C19—C20	−179.5 (4)
Cl1—Cu2—N7—N8	−34.4 (3)	Br4—C18—C19—C20	−0.3 (6)
C17—N7—N8—C19	0.2 (4)	N10—N9—C22—C23	−0.3 (5)
Cu2—N7—N8—C19	167.1 (3)	Cu2—N9—C22—C23	168.5 (3)
N7—Cu2—N9—C22	58.1 (4)	N10—N9—C22—C21	178.9 (4)
Cl3—Cu2—N9—C22	−149.5 (4)	Cu2—N9—C22—C21	−12.2 (7)
Cl1—Cu2—N9—C22	−46.3 (4)	N9—C22—C23—C24	0.7 (5)
N7—Cu2—N9—N10	−134.5 (3)	C21—C22—C23—C24	−178.5 (4)
Cl3—Cu2—N9—N10	17.9 (3)	N9—C22—C23—Br5	−178.7 (3)
Cl1—Cu2—N9—N10	121.0 (3)	C21—C22—C23—Br5	2.0 (7)
C22—N9—N10—C24	−0.2 (5)	N9—N10—C24—C23	0.7 (5)
Cu2—N9—N10—C24	−170.5 (3)	N9—N10—C24—C25	179.4 (4)
N2—N1—C2—C3	−0.1 (5)	C22—C23—C24—N10	−0.8 (5)
Cu1—N1—C2—C3	−172.5 (3)	Br5—C23—C24—N10	178.6 (3)
N2—N1—C2—C1	−179.8 (3)	C22—C23—C24—C25	−179.4 (5)
Cu1—N1—C2—C1	7.9 (6)	Br5—C23—C24—C25	0.1 (8)

### Hydrogen-bond geometry (Å, º)

**Table:**

*D*—H···*A*	*D*—H	H···*A*	*D*···*A*	*D*—H···*A*
N4—H4*N*···Cl3^i^	0.88	2.36	3.194 (4)	160
N10—H10*N*···Cl2^ii^	0.88	2.33	3.144 (3)	155
N2—H2*N*···Cl3	0.88	2.54	3.400 (4)	165
N8—H8*N*···Cl2	0.88	2.34	3.212 (3)	170

Symmetry codes: (i) *x*+1, *y*, *z*; (ii) *x*−1, *y*, *z*.
